# Minimally Invasive Surgical Strategies for the Treatment of Atrial Fibrillation: An Evolving Role in Contemporary Cardiac Surgery

**DOI:** 10.3390/jcdd12080289

**Published:** 2025-07-29

**Authors:** Luciana Benvegnù, Giorgia Cibin, Fabiola Perrone, Vincenzo Tarzia, Augusto D’Onofrio, Giovanni Battista Luciani, Gino Gerosa, Francesco Onorati

**Affiliations:** 1 Cardiac Surgery Unit, Ospedale Santa Chiara di Trento, 38122 Trento, Italy; luciana.benvegnu@apss.tn.it; 2 Cardiac Surgery Unit, Azienda Ospedale, Università di Padova, 35122 Padova, Italy; giorgia.cibin@aopd.veneto.it (G.C.); vincenzo.tarzia@unipd.it (V.T.); gino.gerosa@unipd.it (G.G.); 3 Cardiac Surgery Unit, Azienda Ospedaliera Integrata Verona, 37126 Verona, Italy; perronefabiola5@gmail.com (F.P.); giovanni.luciani@univr.it (G.B.L.); 4 Cardiac Surgery Unit, University of Rome “Tor Vergata”, 00133 Rome, Italy; adonofrio@hotmail.it

**Keywords:** atrial fibrillation ablation, minimally invasive surgery, surgical rhythm control

## Abstract

Atrial fibrillation remains the most frequent sustained arrhythmia, particularly in the elderly population, and is associated with increased risks of stroke, heart failure, and reduced quality of life. While catheter ablation is widely used for rhythm control, its efficacy is limited in persistent and long-standing atrial fibrillation. Over the past two decades, minimally invasive surgical strategies have emerged as effective alternatives, aiming to replicate the success of the Cox-Maze procedure while reducing surgical trauma. This overview critically summarizes the current minimally invasive techniques available for atrial fibrillation treatment, including mini-thoracotomy ablation, thoracoscopic ablation, and hybrid procedures such as the convergent approach. These methods offer the potential for durable sinus rhythm restoration by enabling direct visualization, transmural lesion creation, and left atrial appendage exclusion, with lower perioperative morbidity compared to traditional open surgery. The choice of energy source plays a key role in lesion efficacy and safety. Particular attention is given to the technical steps of each procedure, patient selection criteria, and the role of left atrial appendage closure in stroke prevention. Hybrid strategies, which combine epicardial surgical ablation with endocardial catheter-based procedures, have shown encouraging outcomes in patients with refractory or long-standing atrial fibrillation. Despite the steep learning curve, minimally invasive techniques provide significant benefits in terms of recovery time, reduced hospital stay, and fewer complications. As evidence continues to evolve, these approaches represent a key advancement in the surgical management of atrial fibrillation, deserving integration into contemporary treatment algorithms and multidisciplinary heart team planning.

## 1. Introduction

Atrial fibrillation (AF) is the most common persistent arrhythmia in the general population, with a prevalence of approximately 2–3%, which increases significantly with age, reaching up to 15% in individuals over 80 years of age [1]. The development of AF is closely associated with the presence of atrial fibrosis, both reactive and infiltrative in nature [2]. Recent evidence has also implicated oxidative stress and systemic inflammation as important contributors to atrial remodeling, through their impact on ion channel function and action potential propagation [3,4]. Additional risk factors include genetic predisposition, advanced age, male sex, race, and modifiable lifestyle elements such as smoking, caffeine intake, and sedentary behavior [5].

AF is linked to an elevated risk of heart failure and thromboembolic complications, especially in cases of asymptomatic, or “silent”, AF [6]. Even in patients with early detection, the need for lifelong anticoagulation exposes them to the dual risks of thrombosis and bleeding, significantly affecting quality of life [7].

Over time, various surgical strategies have been developed to manage AF, with the Cox-Maze procedure and its modifications remaining the gold standard. This technique creates a set of atrial lesions designed to block abnormal electrical conduction while preserving sinus rhythm (SR). However, although it is not the only feasible approach, the procedure typically requires a sternotomy to provide more straightforward access to the heart and remains technically complex, particularly in achieving transmural lesions and in the necessity to isolate the posterior wall of the left atrium [8].

In response to the rising prevalence of AF, even in patients without concomitant structural heart disease, minimally invasive surgical approaches have developed. These techniques offer the potential to achieve effective rhythm control while avoiding the morbidity of sternotomy, promoting faster recovery and larger applicability.

The aim of this overview is to critically summarize current minimally invasive surgical strategies for the treatment of atrial fibrillation, highlighting their technical principles and potential advantages.

## 2. Patient Selection and Indications

According to both European and United States guidelines, catheter ablation is an established therapeutic option for patients with symptomatic paroxysmal or persistent AF who remain symptomatic despite optimal treatment with antiarrhythmic drugs.

When considering surgical strategies for AF management, some differences between the guidelines emerge.

The United States guidelines [9] consider surgical ablation and assign a Class IIa recommendation, only in cases of concomitant surgical ablation during other cardiac surgeries. In contrast, the European guidelines provide a class I recommendation (level A evidence) for surgical AF ablation during mitral valve surgery, aiming to reduce AF-related symptoms and decrease the risk of recurrence.

The European guidelines [10] also acknowledge minimally invasive and hybrid AF ablation as valid therapeutic alternatives. These approaches should be considered (Class IIa, Level of Evidence A) in patients with persistent AF refractory to antiarrhythmic drugs, and may be considered (Class IIb, Level of Evidence B) in patients with paroxysmal AF who remain symptomatic despite pharmacologic therapy and have failed previous transcatheter ablation procedures. In all cases, and throughout the patient selection process, decision-making should be conducted by a multidisciplinary heart team comprising experts in cardiology, electrophysiology, anesthesiology, and cardiac surgery.

As is well known, minimally invasive surgery is associated with a harder learning curve and a specific operative setting that requires a high initial investment; however, it offers the advantages of reduced surgical trauma, faster patient recovery, lower incidence of wound dehiscence, and a reduction in overall costs.

Preoperative transesophageal echocardiography and computed tomography (CT) scanning are mandatory for accurate and precise patient management. Particularly, transesophageal echocardiography is essential in the preoperative evaluation, as it provides superior sensitivity compared to transthoracic echocardiography in detecting left atrial appendage thrombi, which contraindicate the procedure due to the risk of embolic events; moreover, it offers detailed anatomical information critical for procedural planning, including assessment of the left atrial appendage, atrial wall thickness, and pulmonary vein anatomy.

Certain anatomical and patient-specific factors may serve as predictors of AF ablation failure. The presence of a severely dilated left atrium (LA) is often associated with failure, especially when patients have an advanced age, concomitant heart failure, and long-standing atrial fibrillation. In particular, prolonged AF raises structural remodeling and the accumulation of fibrosis, both of which significantly contribute to electrophysiological disturbances that ultimately reduce the success rate of the ablation procedure.

## 3. Technologies

The evolution of surgical strategies for AF has been closely supported by the growth in ablation technologies, where the choice of energy source has significantly influenced procedural efficacy and safety. Over the years, several energy modalities have been introduced into clinical practice to replace the incisional lesions of the original Cox-Maze procedure in order to reduce bleeding complications (Table 1) [11]. While radiofrequency (RF) and cryoablation remain the only energy sources approved for surgical ablation in the United States, in Europe, additional technologies such as high-intensity focused ultrasound and microwave energy have been explored, although their clinical use remains limited or under investigation [12].

Focusing not only on new technologies related to the energy sources used, left atrial appendage (LAA) closure represents an important aspect to consider in the context of reducing the cardioembolic risk associated with AF treatment.

LAA closure can be performed either as a stand-alone procedure or adjunctively during AF surgery, depending on the clinical context. As a stand-alone intervention, LAA occlusion is primarily indicated in patients with non-valvular AF who are at high thromboembolic risk and have contraindications to long-term oral anticoagulation.

Thanks to the LAAOS II and III studies [13,14], we now know that left atrial appendage closure performed concomitantly with cardiac surgery not only does not increase operative risk, but more importantly, when combined with anticoagulant therapy, it results in a greater reduction in stroke risk compared to anticoagulant therapy alone.

Finally, closure of the LAA has proven to play a significant role in improving the outcomes of AF ablation, not only by reducing thromboembolic risk, but also by targeting non-pulmonary vein triggers in selected patient cohorts, establishing itself as playing a key role in the management of atrial fibrillation. Clarifying these indications is essential to guide patient selection and optimize therapeutic benefit.

In the context of minimally invasive procedures, LAA occlusion can be performed through a variety of minimally invasive approaches, including mini-thoracotomy, thoracoscopic, and robotic-assisted techniques; in these settings, closure is typically achieved using one of the currently available dedicated devices.

The AtriClip (AtriCure Inc., Mason, OH, USA) is specifically designed for epicardial exclusion of the LAA by occluding its base after direct measurement of the appendage with a specific sizer. Initially developed for use during concomitant cardiac surgery, it has since been adopted for stand-alone LAA closure, particularly in patients with contraindications to long-term anticoagulation therapy, or in combination with procedures for the surgical treatment of AF [15].

The LARIAT device (SentreHeart, Redwood City, CA, USA) represents a more recent and alternative option for LAA exclusion via a minimally invasive approach. Device placement requires not only access to the pericardial cavity, but also the insertion of a magnet-tipped guidewire via femoral venous access with trans-septal puncture. This enables proper positioning of the suture loop at the base of the left atrial appendage, under transesophageal echocardiographic and fluoroscopic guidance [16]. In selected cases where trans-septal puncture is not feasible, the device can be deployed under direct visualization without the need for pre-procedural sizing. This strategy may also be integrated with other procedures, such as the NeoChord mitral valve repair, due to the facilitated access to the LAA, avoiding the need for septal puncture [17].

While its efficacy has been demonstrated [18], larger prospective studies with extended follow-up are warranted to confirm long-term outcomes.

LAA closure is, of course, associated with risks. Malpositioning of the devices can lead to rupture or obstruction of structures adjacent to the AtriClip deployment site, such as the left atrium and the left coronary artery. Regarding the LARIAT device, complications more frequently involve injuries at the peripheral access site or at the level of the left atrium.

## 4. Minimally Invasive Surgical Techniques

Table 2 summarizes and compares the minimally invasive procedures currently available for the surgical treatment of AF.

### 4.1. Mini-Thoracotomy Ablation

Through a unilateral right mini-thoracotomy at the level of the fourth intercostal space, AF ablation can be performed in conjunction with other cardiac procedures, particularly minimally invasive mitral valve surgery. In such cases, femoral vessel cannulation is required to establish cardiopulmonary bypass (CPB). Following a 5–6 mm incision in the fourth intercostal space, the right lung is deflated to improve visualization of the left atrium. After aortic cross-clamping, the left atrium is opened, and the mitral valve procedure is carried out. At this stage, endocardial ablation using cryo energy is performed. Upon closure of the left atrium, RF ablation of the right pulmonary veins may be performed.

This surgical approach is also applicable for stand-alone AF treatment, both with and without the use of CPB. In this setting, the exposure provided is optimal for performing both AF ablation and LAA exclusion. Ablation within the right atrium is generally not performed due to technical challenges in creating the lesion sets required for effective ablation, and more importantly, because of the high risk of subsequent pacemaker implantation.

Since 2005 [19], bilateral thoracotomy has emerged as a valid approach for the treatment of stand-alone AF through the isolation of both right and left pulmonary veins (PVI). Over the years, several modifications to the original technique have been introduced to extend the ablation beyond the pulmonary veins to additional regions of the left and right atria, with the aim of improving the restoration and maintenance of SR.

### 4.2. Thoracoscopic Ablation

Thoracoscopic ablation represents a less invasive modification of the bilateral thoracotomy approach. The primary goal is to achieve PVI and perform RF ablation. This technique also allows for ablation of the posterior wall of the LA and occlusion of the LAA.

Following induction of general anesthesia and placement of a double-lumen endotracheal tube to allow one-lung ventilation, thoracoscopic ports are inserted bilaterally at the level of the mid-axillary line in the fourth and sixth intercostal spaces. The procedure has been well described in the literature [20].

The right thoracic cavity is approached first. After identifying the phrenic nerve, the pericardium is opened, and the pulmonary veins are encircled to allow RF ablation. Ablation is typically repeated at least three times to ensure effective lesion transmurality. The posterior wall, both the roof and the floor of the LA, is also ablated with RF to connect the lesions created on both sides. To increase the efficacy of AF ablation, it is possible to target the ganglionated plexi for ablation. These autonomic structures are located within the epicardial fat and contain both sympathetic and parasympathetic nerve fibers, which play a crucial role in modulating the electrophysiological properties of the myocardium [21].

Subsequently, the left thoracic cavity is accessed. The pericardium is similarly opened, and RF ablation of the left pulmonary veins is performed. At this stage, LAA closure can also be made.

### 4.3. Hybrid Approach

The hybrid approach combines thoracoscopic surgical ablation with percutaneous catheter-based techniques to improve the durability of SR restoration in patients with long-standing persistent AF or in those with AF refractory to pharmacological therapy and previous catheter-based interventions. This strategy necessitates close collaboration between cardiac surgeons and electrophysiologists, who must coordinate the procedural timing—whether as a single combined intervention or as staged procedures. The availability of a hybrid operating room is essential to facilitate the simultaneous execution of both components in a safe and controlled setting.

According to the literature [22], a staged approach may be preferable, allowing for a latency period of approximately 30 to 90 days to assess the effectiveness of the epicardial lesions. Following surgical ablation, detailed endocardial electro-anatomical mapping is useful to evaluate lesion effectiveness. This mapping facilitates the identification of non-transmural or incomplete ablation zones within the left atrium, as well as confirmation of the efficacy of the epicardial lines. Areas of low-voltage or residual conduction identified on the endocardial surface may require additional catheter-based ablation to achieve complete electrical isolation.

The main drawback of the staged hybrid strategy is the need for two separate hospital admissions and two procedures under general anesthesia, which may increase patient operatory risk and healthcare resource utilization.

### 4.4. Convergent Procedure

A dedicated focus on the convergent procedure is necessary. This procedure, introduced in 2009 [23], represents a hybrid approach that combines minimally invasive surgical ablation with endocardial catheter-based techniques. The procedure is performed under general anesthesia with systemic anticoagulation to maintain an activated clotting time (ACT) > 250 s throughout the procedure. Surgically, access is realized via a subxiphoid incision of approximately 3–5 cm, with optional removal of the xiphoid process. After opening the pericardium, a temperature probe is placed near the posterior wall of the LA to monitor for potential esophageal thermal injury.

Through the oblique sinus, both a cannula and a thoracoscope are introduced to guarantee optimal visualization of the posterior pericardium and LA wall. An RF ablation device is then passed through the cannula to create epicardial lesions. If an increase in temperature is detected by the esophageal probe, ablation is arrested immediately to avoid collateral tissue damage. To maintain thermal safety, continuous irrigation with cold saline solution is applied during the ablation process [24,25]. A further advancement is represented by robotic-enhanced ablation, an evolution of the thoracoscopic convergent approach. This technique utilizes the same equipment but allows the procedure to be performed without the need for a subxiphoid access [26].

Typically, 20–30 lesions are created to ensure the effectiveness and durability of the ablation. At the conclusion of the procedure, a pericardial drain is placed to monitor for postoperative bleeding and is generally removed after 24 h if no complications arise.

As supported by current evidence [14], concomitant closure of the LAA during epicardial ablation significantly reduces the risk of stroke and should be considered whenever feasible. Preoperative transesophageal echocardiography is essential to exclude the presence of thrombus within the LAA prior to intervention.

The endocardial component of the procedure is typically performed in the same session. Access to the left atrium is obtained via a femoral vein puncture followed by a trans-septal approach, guided by both fluoroscopy and echocardiography. This allows for mapping and potential ablation of residual arrhythmogenic foci not addressed during the epicardial phase.

## 5. Conclusions

The evolution of minimally invasive surgical strategies for AF reflects the increasing demand for effective, less traumatic alternatives to traditional open-heart procedures. Techniques such as thoracoscopic ablation, mini-thoracotomy approaches, and hybrid procedures, including the convergent strategy, offer valuable treatment options for patients with persistent or long-standing AF, especially those who are unresponsive to antiarrhythmic drugs or catheter-based interventions. These approaches allow for posterior wall isolation and LAA exclusion while minimizing surgical trauma, helping with faster recovery and reducing perioperative morbidity. Despite these advantages, limitations remain, including variability in realizing transmural lesions, the need for advanced technical expertise, and the requirement for multidisciplinary coordination, particularly in hybrid settings. Another important limitation is the partial availability of long-term data regarding the durability of these strategies, particularly in terms of sustained freedom from atrial fibrillation. The heterogeneity in patient selection, procedural techniques, and follow-up protocols across centers makes it challenging to draw definitive conclusions about their long-term efficacy. Additionally, the management of rhythm follow-up in the context of hybrid procedures presents logistical and clinical challenges. Ensuring seamless coordination between electrophysiologists and cardiac surgeons remains essential but can be difficult to implement consistently. A truly multidisciplinary approach is required not only during the index procedure but throughout the follow-up period to optimize outcomes, manage recurrences, and tailor subsequent interventions. Future research efforts and collaborative models are needed to address these limitations and to establish standardized protocols that can enhance both the clinical effectiveness and organizational efficiency of these promising strategies.

Focusing on the literature, thoracoscopic ablation typically demonstrates sinus rhythm maintenance rates of approximately 68–80% at 5–7 years, with relatively low complication rates [27,28]. The hybrid convergent approach, combining epicardial and endocardial ablation, has been associated with similar long-term efficacy (freedom from atrial arrhythmias ranging from 75–80% at 1–3 years), particularly in persistent and long-standing persistent AF, though it carries slightly increased periprocedural risks due to its dual-access nature [29,30]. Robotic-assisted approaches offer enhanced precision and reduced invasiveness, though data on long-term rhythm outcomes remain limited due to the novelty of the technique and smaller case volumes. Regarding safety, stroke incidence across all approaches remains low (<2%), largely due to concomitant left atrial appendage management and perioperative anticoagulation protocols. Complication profiles vary with the approach used, with thoracoscopic methods generally associated with lower morbidity but potentially reduced efficacy in complex AF. Overall, while all strategies show acceptable safety and effectiveness, definitive conclusions are not possible due to heterogeneity in patient selection, follow-up duration, and rhythm monitoring protocols across studies. High-quality, randomized comparative data remain limited and are needed to define the optimal strategy tailored to patient-specific factors.

To date, the treatment of atrial fibrillation combined with other cardiac surgeries remains infrequent (less than 50% eligible for AF ablation receive the treatment) and should certainly be encouraged. An interesting aspect is that the treatment of atrial fibrillation is closely associated with the surgeon’s experience, likely because it is still perceived as surgically riskier, even though evidence has shown that this is not the case [31]. Based on the available literature, it has been shown that treating atrial fibrillation during cardiac surgery improves freedom from atrial fibrillation compared to patients who are not treated. However, there is no conclusive evidence that restoration of sinus rhythm translates into a reduction in mortality or cardioembolic events compared to untreated patients [32,33]. In this context, further studies with longer follow-up are warranted to better clarify these outcomes.

While the hybrid and convergent approaches are well-characterized in terms of procedural technique, a comparative evaluation with stand-alone surgical and catheter-based strategies reveals important distinctions in clinical outcomes and procedural considerations. Hybrid and convergent procedures aim to combine the durability of surgical epicardial ablation, particularly for posterior wall isolation, with the precision and mapping abilities of endocardial catheter ablation. This dual approach has been shown to improve long-term sinus rhythm maintenance, especially in patients with persistent or long-standing persistent AF, where catheter ablation alone often produces suboptimal outcomes. In contrast, stand-alone thoracoscopic surgical ablation, though effective in realizing transmural lesions, may lack real-time mapping and carry increased perioperative morbidity. Conversely, catheter-based ablation offers a less invasive option with reduced recovery time, but recurrence rates remain higher, particularly in advanced AF substrates. Therefore, while hybrid strategies may involve greater procedural complexity and coordination among multidisciplinary teams, they potentially offer superior arrhythmia control in selected high-risk patients. Further comparative studies and randomized trials are warranted to refine patient selection and determine the most effective approach based on individual clinical profiles.

Pharmacological therapy plays a crucial adjunctive role in supporting procedural success after AF ablation, particularly in maintaining long-term sinus rhythm. While traditional antiarrhythmic drugs remain a keystone in early post-procedural management, there is growing interest in agents that may favorably influence atrial remodeling. Among these, sodium–glucose cotransporter 2 inhibitors (SGLT2is), originally developed for glycemic control in type 2 diabetes, have demonstrated promising cardiovascular effects. Recent preclinical and clinical evidence [34] suggests that SGLT2is may reduce AF burden and atrial arrhythmogenic substrate by attenuating oxidative stress, inflammation, and atrial fibrosis. These pathophysiological mechanisms are intimately involved in the progression and maintenance of AF, and their modulation could increase the durability of rhythm control strategies, including catheter or surgical ablation. In patients with metabolic comorbidities—frequently present in those undergoing AF surgery—the incorporation of SGLT2is into the therapeutic regimen may offer benefits. Therefore, the potential disease-modifying effects of SGLT2 inhibitors deserve further investigation in the context of AF ablation. Incorporating such agents into postoperative management protocols may represent a novel and promising approach to improving long-term procedural outcomes.

Future perspectives focus on refining lesion delivery technologies, improving intraoperative lesion assessment, and enhancing real-time imaging and mapping tools. Novel energy sources, such as pulsed field ablation (PFA), and integration of artificial intelligence in mapping and decision-making may help optimize outcomes and procedural efficiency. PFA utilizes high-voltage, microsecond-duration electrical pulses to induce irreversible electroporation, selectively targeting myocardial tissue while saving adjacent structures. This tissue specificity may translate into improved safety profiles, especially in anatomically complex regions near the esophagus, phrenic nerve, or pulmonary veins. Early clinical studies, including the IMPULSE, PEFCAT, and PEFCAT II trials, have demonstrated encouraging acute efficacy and low complication rates, supporting the feasibility of PFA in both endocardial and epicardial settings [35]. From a surgical standpoint, the adoption of PFA could enhance the safety and efficiency of hybrid or stand-alone thoracoscopic ablation procedures, potentially reducing operative times and improving lesion consistency. Given these advantages, a more detailed consideration of PFA within the context of minimally invasive surgical approaches may be warranted. Its future incorporation into surgical plans could represent a paradigm shift in the treatment of AF, deserving close attention as clinical evidence continues to evolve.

Additionally, the development of standardized protocols and patient-specific treatment algorithms, guided by electrophysiological, anatomical, and imaging data, could further personalize surgical AF treatment. Long-term data from randomized controlled trials comparing minimally invasive surgical techniques with catheter ablation and conventional surgery will be essential to better define indications, timing, and outcome predictors.

In conclusion, minimally invasive surgical ablation is a rapidly advancing field that holds significant promise within a comprehensive, multidisciplinary rhythm management strategy. Continued innovation and collaboration across specialties will be key to maximizing its potential and improving long-term patient outcomes.

## Figures and Tables

**Table 1:** Comparison of energy sources used in surgical atrial fibrillation ablation.

Energy Source	Mechanism of Action	Tissue Penetration	Advantages	Limitations
Radiofrequency (RF)	Resistive heating via alternating current	3–5 mm	Well-established, transmurality can be monitored, available in unipolar and bipolar forms	Risk of charring, incomplete lesions in thick atrial tissue, esophageal and coronary artery injury
Cryoablation	Freezing leads to ice crystal formation and cellular disruption	4–6 mm	Preserves tissue architecture, less thrombogenic, good for pulmonary vein isolation	Longer application time, risk of incomplete lesions in warm areas
High-Intensity Focused Ultrasound	Acoustic energy generates heat at focal point	3–6 mm	Precise, no direct tissue contact needed, lower collateral damage	Limited availability, risk of esophageal injury
Microwave	Electromagnetic radiation causes molecular agitation and heat	3–10 mm	Can create wide, continuous lesions	Less controlled lesion size, possible collateral damage
Laser	Focused light energy converted into heat	2–4 mm	High precision, minimal collateral damage	Expensive, limited penetration depth, less widespread use

**Table 2:** Comparative overview of minimally invasive surgical techniques for atrial fibrillation.

Technique	Surgical Approach	Energy Source	Advantages	Limitations
Mini-Thoracotomy Ablation	Left mini-thoracotomy (anterior axillary line)	Bipolar RF/Cryoablation	Direct access to LA, compatible with LAA exclusion, good transmurality	Unilateral access, less flexible for biatrial lesions
Thoracoscopic Ablation	Bilateral thoracoscopic access	Bipolar RF/Cryoablation	No sternotomy, good visualization, pulmonary vein isolation (PVI)	Technically demanding, requires single-lung ventilation, limited access to LA
Hybrid Approach (Surgical + EP)	Thoracoscopic + transcatheter (staged or simultaneous)	Bipolar RF/Cryo + Endocardial RF	Comprehensive lesion sets, improved long-term success, tailored per patient	Requires coordination between specialties, multiple procedures
Convergent Procedure	Subxiphoid or limited thoracoscopic + endocardial	Unipolar RF + Endocardial RF	Posterior LA wall ablation, suitable for persistent AF, less invasive	Limited epicardial access, technically demanding, not for all AF types

Legend: RF: radiofrequency; PVI: pulmonary vein isolation; LA: left atrium; EP: electrophysiologist; AF: atrial fibrillation; LAA: left atrial appendage.

## Data Availability

No new data were created or analyzed in this study. Data sharing is not applicable.

## Abbreviations

The following abbreviations are used in this manuscript:

AF	Atrial Fibrillation
CT	Computed Tomography
RF	Radiofrequency
LAA	Left Atrial Appendage
CPB	Cardiopulmonary Bypass
PVI	Pulmonary Vein Isolation
EP	Electrophysiologist
SR	Sinus Rhythm
LA	Left Atrium
ACT	Activated Clotting Time
SGLT2i	Sodium–Glucose Cotransporter 2 Inhibitor
PFA	Pulsed Field Ablation
